# SWATted away: the challenging experience of setting up a programme of SWATs in paediatric trials

**DOI:** 10.1186/s13063-019-3236-4

**Published:** 2019-02-19

**Authors:** Jacqueline Martin-Kerry, Adwoa Parker, Peter Bower, Ian Watt, Shaun Treweek, David Torgerson, Catherine Arundel, Peter Knapp

**Affiliations:** 1 Department of Health Sciences, University of York, Heslington, York, YO10 5DD UK; 2 MRC North West Hub for Trials Methodology Research, NIHR School for Primary Care Research, University of Manchester, Manchester, M13 9PL UK; 3 Department of Health Sciences and the Hull York Medical School, University of York, Heslington, York, YO10 5DD UK; 4 Health Services Research Unit, University of Aberdeen, Foresterhill, Aberdeen, AB25 2ZD UK

**Keywords:** ‘SStudy Within A Trial’ (SWAT), embedded trials, methodology, challenges, randomised controlled trials, paediatrics, governance

## Abstract

**Background:**

Randomised controlled trials are considered the best method for determining the effectiveness and safety of health interventions. Trials involving children are essential to ensure that treatments are safe and effective. However, many trials, in both adult and paediatric populations, do not achieve recruitment targets and/or maintain retention of participants, which can lead to a reduction in the internal and external validity of the results. Identifying ways of improving trial efficiency are important in order to increase the successful completion of trials.

**Main body:**

A ‘Study Within A Trial’ (SWAT) is a self-contained study embedded within an ongoing trial, which aims to establish evidence to improve the management and delivery of trials in healthcare. Increasing numbers of SWATs have been undertaken in recent years yet very few within paediatric trials. Herein, we describe some of the challenges with undertaking a programme of SWATs within paediatric clinical trials in the UK. The TRECA (TRials Engagement in Children and Adolescents) study involves developing multimedia websites for use within paediatric trials to provide recruitment information to children, young people and their families about the clinical trial. Challenges encountered included governance issues such as host trial approval processes and sharing of anonymised data, funding issues for host trials, internet quality and accessibility within the healthcare setting, and ethical concerns associated with SWAT methodology. We believe the ethical concerns are more pronounced in the paediatric setting, perhaps because of the fewer SWATs undertaken in these settings or that a more cautious, risk-averse approach to undertaking research with children is taken.

**Conclusion:**

SWATs are becoming increasingly common to provide an evidence base for methods to improve trial efficiency. However, we encountered a number of unanticipated challenges to embedding TRECA that have not been previously reported within the scientific literature. We believe that, if these issues were addressed through wider promotion and explanation of undertaking SWATs involving all key stakeholders, as well as by exploration of alternative funding models for SWATs, this would enable more streamlined, appropriate and timely processes for SWATs and a stronger evidence base for what works to increase trial efficiency.

**Trial registration:**

The TRECA study is registered on ISRCTN, ID 73136092. Registered on 24 August 2016.

## Background

### The need for evidence-informed trials

Although randomised controlled trials (RCTs) are the gold standard for developing an evidence base on the effectiveness of healthcare interventions, significant uncertainties exist about their design, conduct and reporting, meaning that trials are often not efficient. For example, approximately 50% of trials fail to achieve their original recruitment targets [1]. Poor recruitment and retention of trial participants can be very costly [2] and contribute significantly to research waste [3, 4].

The UK government has highlighted its ambition to accelerate the development of innovative medicines to improve patient health outcomes and healthcare efficiency [5]. However, without the ability to accelerate the evaluation of healthcare innovations, and for these evaluations to be completed in time and to target, this ambition will be stymied. Furthermore, despite our focus on the UK, this issue is faced by many health systems around the world.

### ‘Study Within A Trial’ (SWAT), an emerging field

With the recognition that developing the evidence base for trials should be a priority, there has been a recent international movement to improve the efficiency and successful delivery of trials through the use of rigorous evaluation, adopting the SWAT methodology. A SWAT is a “*self-contained study that has been embedded within a host trial with the aim of evaluating or exploring alternative ways of delivering or organising a particular trial process*” [6]. For instance, in the UK, the Medical Research Council funded the START (Systematic Techniques for Assisting Recruitment to Trials) programme, which successfully developed a conceptual, methodological and logistical framework to improve recruitment through embedding SWATs of recruitment interventions in multiple host trials, and developed reporting guidelines for recruitment SWATs [7, 8]. The Northern Ireland Hub for Trials Methodology Research has established the SWAT Repository to facilitate SWATs [9]. Trial Forge is another UK initiative, based in Scotland, that aims to increase the evidence base for trial decision-making and, in doing so, improve trial efficiency. Trial Forge recently published guidance for defining a SWAT [6]. The current Medical Research Council-funded PROMoting THE USE of SWATs (PROMETHEUS) programme [10] is building on the START initiative to make SWATs standard practice in clinical trials in the UK by funding and facilitating the initiation of at least 25 SWATs across multiple teams in the UK. Recently, the UK National Institute for Health Research (NIHR) announced a new funding stream for SWATs in the Health Technology Assessment (HTA) programme [11], which has the potential to increase the number of trial teams likely to consider and/or actively undertake SWATs. In the Republic of Ireland, the Health Research Board – Trials Methodology Research Network, supports and funds research teams to undertake SWATs to improve the efficient conduct of future trials [12].

### Previously identified challenges with SWATs

Despite the current focus on SWATs, a range of challenges to undertaking them have been identified. Challenges for host trials include increased complexity and management burden, compatibility between the host and embedded trials, and the impact of the embedded trial on host trial design and relationships with collaborators [13]. For embedded trials, there are concerns that host trial investigators might have strong preferences, limiting the control that embedded study investigators have over their research, and that inadequate sample sizes may limit statistical power [13]. Other identified challenges include cost, resistance of the chief investigator or co-investigators, funding for SWATs, and distraction and additional workload for research staff [14, 15].

### The TRECA Study, an example of a SWAT to evaluate a new recruitment intervention

In this paper, we discuss some of the challenges encountered within a programme of SWATs, the TRials Engagement in Children and Adolescents (TRECA) Study [16], funded by the UK NIHR Health Services and Delivery Research Programme (14/21/21). TRECA is investigating a novel alternative to a printed participant information sheet (PIS) for children, young people and their parents, when approached about a clinical trial. This is an important opportunity to explore alternative methods of providing information as many PIS documents are lengthy, difficult to understand and do not incorporate visual elements [17–20]. In the first phase of the TRECA study, multimedia website templates about paediatric clinical trials using text, pictures, animations and short video clips were developed [21] and user tested [22]. Phase two of TRECA began in late 2017 and involves adapting the multimedia websites for six paediatric clinical trials (host trials) using trial-specific content and embedding the websites as recruitment tools within the host trials. There is a lack of evidence on the effectiveness of multimedia for supporting decision-making about trials, particularly in the paediatric setting. When host trials embed TRECA, the trial randomises those approached about trial participation to one of three arms of TRECA so that each person approached receives one of the following: the PIS only, the multimedia website only, or both the PIS and multimedia website. We are interested in the impact of the multimedia websites on the recruitment and retention rates to the six trials, as well as the quality of decision-making by families about trial participation.

Despite much interest and enthusiasm for SWATs, and clear benefits for utilising them to evaluate new methodological interventions within RCTs [6], we have encountered a number of challenges to embedding TRECA within UK paediatric trials. Herein, we describe these challenges and suggest some possible solutions that may enable SWATs to be undertaken more quickly and efficiently within a paediatric context, or other settings where there is a perception of patient vulnerability or risk.

## Challenges faced by TRECA

The main challenges encountered when engaging with potential host trials to embed TRECA fall under four main categories, namely governance and approvals, funding;, methodological/ethical concerns, and internet access and quality.

### Governance and approvals issues

A number of governance and approvals issues have been encountered when embedding TRECA within host paediatric trials. Within Phase two of TRECA, each of the six host trials had different approval processes to embed TRECA. Some trials required their Trial Management Group (TMG) to formally approve collaboration. Other host trials requested that a feasibility questionnaire be developed by TRECA and sent to all potential host trial sites. The questionnaires were accompanied by information about TRECA in terms of the practicalities of what would be involved if the host trial site was to embed TRECA. We sought each site’s approval and agreement with embedding TRECA through the completion of a set of questions relating to the process of embedding TRECA. From this, the decision still rested with the TMG, which may have only met infrequently. One host trial required two sets of feasibility questionnaires to be circulated to the trial sites – one prior to a decision by the host TMG about embedding TRECA, and another following this decision. In our experience, it often took 3–8 months from initial discussions with the potential host trial until the trial made a decision about embedding TRECA; this had an important time-delaying impact on TRECA’s timelines. Crucially, TRECA could not begin developing the multimedia websites (given they are tailored to the trial) until the decision was made by the host trial, and the delay then impacted on the development and embedding of the websites (the tested recruitment intervention).

So that TRECA could evaluate the impact of the multimedia websites on recruitment, retention and quality of decision-making, we required anonymised patient data from each host trial. To this end, we developed a data sharing agreement. Whilst we expected that these agreements would be straightforward, host trial sponsors raised concerns about sharing even anonymised data, and legal teams from the host trials’ sponsors reviewed and queried the agreements prior to signing. In addition, recent changes in data protection with the recent General Data Protection Regulation and Data Protection Act 2018 also led to further concerns about sharing of anonymised data and the need for a transparent approach to informing participants about the sharing of their data between organisations. One host trial noted that the sponsor of the host trial would not be signing the agreement, and instead required each participating host trial site to sign an individual data-sharing agreement with TRECA, increasing the administration and workload substantially.

### Funding issues for trials embedding SWATs

Another challenge encountered relates to funding. The NIHR Clinical Research Network (CRN) provides funding to trials in the UK through the process of funding per participant recruited (accruals) for so-called ‘portfolio-adopted’ research studies. The Portfolio comprises high quality clinical research studies that are eligible for CRN funding and support. Recruitment data allows the allocation of funding to the NIHR Local CRNs to direct National Health Service (NHS) support to sites. Almost every trial we have approached about TRECA has asked or assumed that the host trial would receive two sets of accruals – one for recruitment of their participants into the host trial, and the second for those who were randomised to TRECA. However, the CRN considers this situation to be ‘double-counting’ as all of those recruited to the host trial would have been approached using one of the arms of TRECA and an additional consent process for the SWAT is not required. Nevertheless, we can see the trial’s view that by embedding TRECA they are introducing a greater workload, although the TRECA team aims to reduce this burden as much as is practicable. Receiving additional funding for the local CRN may provide an incentive for a trial to embed a SWAT, particularly for the recruiters, as this funding may enable the CRN to support the trial team.

Another accrual issue relates to a potential host trial for TRECA that was not portfolio adopted. This particular host trial team thought that by embedding TRECA, which is an NIHR portfolio-adopted study, they would then be able to access an NIHR research nurse through funding/accruals to undertake recruitment for the host trial. However, this was not possible under the current CRN process. This raises the question of whether another funding model would assist with recruiting trials to undertake SWATs. A middle ground may be to provide a recruitment incentive for trials to undertake SWATs but below the level of accrual/funding for recruiting a trial participant. Another option is to utilise the PROMETHEUS [10] model (https://www.york.ac.uk/healthsciences/research/trials/research/swats/prometheus/) with a flat rate for a SWAT provided to the trial team.

### Confusion around embedded trial methodology and ethical concerns

Trialists have often been unsure about the methodology and approvals of embedded trials. We sought overarching research ethics and Health Research Authority (HRA) approvals for TRECA prior to identifying and approaching potential host trials. In this overarching ethics application, we sought (and received) approval so that host trials do not need to explain TRECA or seek consent for those approached about the host trial in order to be randomised within TRECA. This is because explaining TRECA to those approached about the host trial and seeking consent to TRECA would be confusing and may also confound the effect of the information intervention being tested in the SWAT. However, trials have generally expressed concern about people not needing to consent to the embedded trial, despite these concerns not being raised by research ethics committees or the HRA.

In addition, NHS Trust Research & Development (R&D) departments (located within NHS sites and responsible for granting approval for research studies being undertaken locally) are often unclear of how to review and approve embedded trials, which causes delays. For example, one trial initially reviewed the TRECA documentation as an embedded study and then decided that TRECA would be reviewed as a stand-alone study, subsequently requesting all documentation to be sent again and reviewed. In addition, R&D departments were often unsure about which documentation they needed to review and some had concerns about participants not consenting to the SWAT (despite ethics approval for this process). These additional steps caused further delays in embedding TRECA.

### Accessibility and quality of internet provision

An unexpected challenge with undertaking a SWAT involving the delivery of a multimedia website within the healthcare setting was the variation in Wi-Fi network conditions and permissions at each NHS site. This proved challenging when developing the multimedia websites for host trials as the Principal Investigator for one host trial was unable to view the websites due to internet viewing restrictions at the hospital (the videos and animations are stored on a site which was blocked at this particular hospital). Furthermore, some Wi-Fi networks were either too slow to load animations and videos or could not be reliably accessed. We overcame this issue by providing affected sites with a tablet computer that had an internet SIM card.

## Other learnings from the TRECA study

Despite the challenges we faced with incorporating this programme of SWATs within six host trials, we encountered a number of positive experiences. There is a genuine interest in presenting information about trials to families in a more engaging way and there has been a great deal of enthusiasm for the multimedia websites created. We also found Research Ethics Committees and the HRA to be very supportive of us evaluating the use of multimedia websites as an alternative or supplement to printed PIS documents. We also developed a structured and quality method of creating multimedia websites by working with host trials and a company that specialises in developing websites and animation (Morph; www.morph.co.uk). For researchers wanting to implement SWATs in the future, we would recommend early engagement with all stakeholders (including trialists, sponsors, R&D department staff) about incorporating a SWAT so that any concerns or queries are addressed early. We would also factor in a lead time of 6 months for trials to sign the data-sharing agreement.

## Conclusions

SWATs have become increasingly popular, offering an opportunity to identify what works best when undertaking trials [6]. In conducting Phase two of the TRECA study, we identified and described a number of governance, funding and methodological challenges when embedding a programme of SWATs within host paediatric trials. There are a small number of publications describing challenges with embedding SWATs [13–15]; however, some of the issues identified within the TRECA study have not previously been described and this paper provides detailed information about the challenges faced. We also are not aware of any publications about SWATs undertaken within paediatric trials, and believe that some of the challenges we have experienced have a more marked impact in the paediatric context and in other contexts where there is a perception of increased patient vulnerability or risk. For example, a recent Cochrane review showed that only one of 68 trials evaluating strategies to improve recruitment into RCTs had included a paediatric sample [23]. However, we believe that the challenges we have identified within TRECA may be applicable to trials with other populations, including trials involving adults, and are relevant for other researchers wishing to undertake SWATs in a variety of trials and settings. We also acknowledge that the issue of internet quality and access will only impact on SWATs that involve delivery of websites and not on other methods of information provision.

We believe that the identified challenges can be overcome, enabling a more streamlined and proportionate approach to trials reviewing requests for SWATs. We suggest that increasing awareness of SWATs more widely in the UK, such as through publications and presentations, and ensuring that paediatric trialists are involved, would assist with some of the ethical concerns raised, including participants not needing to provide explicit consent for the SWAT. We feel that the ethical concerns expressed by host trials for TRECA reflect that this study was undertaken in the paediatric setting where there may be more caution about novel methods. It is important that all stakeholders are involved in a process of increasing SWAT awareness, including members of ethical committees, sponsor representatives, principal investigators, trial managers and coordinators, TMGs, CRNs, R&D officers, trial managers, and coordinators at trial sites and clinical trial units.

We also feel that the provision of more guidance to NHS sites and trials about how to review a SWAT, and earlier identification of whether the host trial is able to embed it, would be beneficial. Undertaking feasibility assessments with sites participating in a multi-centre trial takes considerable time in order to develop and distribute the questionnaire, answer site queries, collate results and then await TMG review. In addition, we have found that a number of R&D departments are not familiar with SWAT methods, how to review SWATs, or the order in which they should review and approve studies (i.e. approval before or after the host trial). R&D departments ultimately approve the undertaking of SWATs at sites and are often not involved in early discussions with trialists about including a SWAT. Ensuring that R&D departments are more familiar with SWATs would streamline the process of incorporation within new and existing trials. If these elements can be addressed, we would hope that this would enable more SWATs to be undertaken, providing a stronger evidence base about what works best in RCTs. In terms of funding models for host trials embedding a SWAT, we feel that alternative models should be explored to generate incentives for host trials that match the workload of undertaking the SWAT, and the HTA funding stream may provide a viable funding alternative. We have described the UK situation but feel that these issues of funding support to host trials may be similar in other countries.

In summary, we suggest that the following actions may overcome some of the challenges with undertaking SWATs in the paediatric setting:

1. Reduce ethical approval and governance barriers by increasing awareness of SWATs and engaging all stakeholders (including ethical committees, sponsor representatives, principal investigators, trial managers and coordinators, TMGs, R&D and trial sites).
2. Provide more guidance and explanation about SWATs. In the UK, this could be led by NIHR or HRA, who are perhaps best positioned to provide the guidance and support.
3. Explore other funding models that may better support SWATs. This may be through a down-weighted recruitment incentive for SWATs through the CRN, or using the PROMETHEUS model of providing a set amount to trial teams for undertaking a SWAT, or using the new HTA funding stream.
4. Review existing internet access in hospitals to determine whether improved access can be enabled to allow interventions such as multimedia websites about trials or healthcare treatments to be accessed more easily.

## Abbreviations

CRN: Clinical Research Network
HRA: Health Research Authority
HTA: Health Technology Assessement
NHS: National Health Service
NIHR: National Institute for Health Research
PIS: participant information sheet
PROMETHEUS: PROMoting THE USE of SWATs
R&D: Research and Development
RCT: randomised controlled trial
START: Systematic Techniques for Assisting Recruitment to Trials
SWAT: Study Within A Trial
TMG: Trial Management Group
TRECA: TRials Engagement in Children and Adolescents

## References

[1] Walters SJ, Bonacho dos Anjos Henriques-Cadby I, Bortolami O, Flight L, Hind D, Jacques RM (2017). Recruitment and retention of participants in randomised controlled trials: a review of trials funded and published by the United Kingdom Health Technology Assessment Programme. BMJ Open.

[2] Kitterman DR, Cheng SK, Dilts DM, Orwoll ES (2011). The prevalence and economic impact of low-enrolling clinical studies at an academic medical center. Acad Med.

[3] Moher D, Glasziou P, Chalmers I, Nasser M, Bossuyt PM, Korevaar DA (2016). Increasing value and reducing waste in biomedical research: who’s listening?. Lancet.

[4] Ioannidis JP, Greenland S, Hlatky MA, Khoury MJ, Macleod MR, Moher D (2014). Increasing value and reducing waste in research design, conduct, and analysis. Lancet.

[5] Department of Health and Wellcome Trust (2016). Accelerated Access Review Final Report: Review of Innovative Medicines and Medical Technologies.

[6] Treweek S, Bevan S, Bower P, Campbell M, Christie J, Clarke M (2018). Trial Forge Guidance 1: what is a Study Within A Trial (SWAT)?. Trials.

[7] Rick J, Graffy J, Knapp P, Small N, Collier DJ, Eldridge S (2014). Systematic techniques for assisting recruitment to trials (START): study protocol for embedded, randomized controlled trials. Trials.

[8] Madurasinghe VW (2016). Guidelines for reporting embedded recruitment trials. Trials.

[9] The Northern Ireland Hub for Trials Methodology Research (2018). SWAT Repository Store..

[10] University of York (2018). PROMoting THE USE of SWATs (PROMETHEUS)..

[11] National Institute of Health (2018). Studies Within A Trial (SWATs).

[12] Health Research Board Trial Methodology Research Network (2018). Study Within A trial (SWAT).

[13] Graffy J, Bower P, Ward E, Wallace P, Delaney B, Kinmonth AL (2010). Trials within trials? Researcher, funder and ethical perspectives on the practicality and acceptability of nesting trials of recruitment methods in existing primary care trials. BMC Med Res Methodol.

[14] Adamson J, Hewitt CE, Torgerson DJ (2015). Producing better evidence on how to improve randomised controlled trials. BMJ.

[15] Rick J, Clarke M, Montgomery AA, Brocklehurst P, Evans R, Bower P (2018). Doing trials within trials: a qualitative study of stakeholder views on barriers and facilitators to the routine adoption of methodology research in clinical trials. Trials.

[16] Martin-Kerry J, Bower P, Young B, Graffy J, Sheridan R, Watt I (2017). Developing and evaluating multimedia information resources to improve engagement of children, adolescents, and their parents with trials (TRECA study): Study protocol for a series of linked randomised controlled trials. Trials.

[17] Caldwell PH, Dans L, de Vries MC, Newman Ba Hons J, Sammons H, Spriggs MBM (2012). Standard 1: consent and recruitment. Pediatrics.

[18] Ogloff JR, Otto RK (1991). Are research participants truly informed? Readability of informed consent forms used in research. Ethics Behav.

[19] Ennis L, Wykes T (2016). Sense and readability: participant information sheets for research studies. Br J Psychiatry.

[20] Franck L, Winter I (2004). Research participant information sheets are difficult to read. Bull Med Ethics..

[21] Martin-Kerry JM, Knapp P, Atkin K, Bower P, Watt I, Stones C et al. Supporting children and young people when making decisions about joining clinical trials: qualitative study to inform multimedia website development. BMJ Open 2019;9:e023984. 10.1136/bmjopen-2018-023984. 10.1136/bmjopen-2018-023984 PMC6340013 30782720

[22] Sheridan R, Martin-Kerry J, Watt I, Higgins S, Stones SR, Taylor DH, et al. User testing digital, multimedia information to inform children, adolescents and their parents about healthcare trials. J Child Health Care. 2018. 10.1177/1367493518807325. 10.1177/1367493518807325 30384772

[23] Treweek S, Pitkethly M, Cook J, Fraser C, Mitchell E, Sullivan F (2018). Strategies to improve recruitment to randomised trials. Cochrane Database Syst Rev.

